# Objective structured assessment ultrasound skill scale for hyomental distance competence – psychometric study

**DOI:** 10.1186/s12909-023-04146-y

**Published:** 2023-03-22

**Authors:** Sara Hora Gomes, Marta Trindade, Cristina Petrisor, Dinis Costa, Jorge Correia-Pinto, Patrício S. Costa, José M. Pêgo

**Affiliations:** 1 Life and Health Sciences Research Institute (ICVS), School of Medicine, University of Minho, Campus Gualtar, Braga, 4710-057 Portugal; 2 ICVS/3B’s-PT Government Associate Laboratory, Braga/Guimarães, 4710-057 Portugal; 3 Anesthesia and Intensive Care II Department, Pharmacy Cluj-Napoca and Anesthesia and Intensive Care Department, “Iuliu Hatieganu” University of Medicine, Clinical Emergency County Hospital, Cluj-Napoca, 400347 Romania; 4 Department of Anesthesia, Hospital de Braga, Braga, 4710-243 Portugal; 5 Department of Pediatric Surgery, Hospital de Braga, Braga, 4710-243 Portugal; 6 iCognitus4ALL – IT Solutions, Braga, 4470-057 Portugal

**Keywords:** OSAUS, Hyomental, Distance, Airway, Ultrasound, Assessment, Psychometric properties

## Abstract

**Background:**

Ultrasound assessment of the airway recently integrates the point-of-care approach to patient evaluation since ultrasound measurements can predict a difficult laryngoscopy and tracheal intubation. Because ultrasonography is performer-dependent, a proper training and assessment tool is needed to increase diagnostic accuracy. An objective, structured assessment ultrasound skill (OSAUS) scale was recently developed to guide training and assess competence. This work aims to study the psychometric properties of OSAUS Scale when used to evaluate competence in ultrasound hyomental distance (HMD) measurement. Methods: Prospective and experimental study. Volunteers were recruited and enrolled in groups with different expertise. Each participant performed three ultrasonographic HMD evaluation. The performance was videorecorded and anonymized. Five assessors blindly rated participants’ performance using OSAUS scale and a Global Rating Scale (GRS). A psychometric study of OSAUS scale as assessment tool for ultrasound HMD competence was done. Results: Fifteen voluntaries participated on the study. Psychometric analysis of OSAUS showed strong internal consistency (Cronbach’s alpha 0.916) and inter-rater reliability (ICC 0.720; p < 0.001). The novice group scored 15.4±0.18 (mean±SD), the intermediate 14.3±0.75 and expert 13.6±0.1.25, with a significant difference between novice and expert groups (p = 0.036). The time in seconds to complete the task was evaluated: novice (90±34) (mean±SD), intermediate (84±23) and experts (83±15), with no significant differences between groups. A strong correlation was observed between OSAUS and global rating scale (r = 0.970, p < 0.001).

**Conclusion:**

The study demonstrated evidence of validity and reliability. Further studies are needed to implement OSAUS scale in the clinical setting for training and assessment of airway ultrasound competence.

## Background

In the last decades, the use of ultrasonography expanded from the imaging laboratory to patient bedside evaluation. Nowadays, almost all medical specialties have developed a Point-of-Care Ultrasonography (PoCUS) approach to enhance patients’ primary assessment, diagnostics and treatment. In anesthesiology training curriculum and daily practice several PoCUS approaches to patient evaluation have been incorporated, namely for cardiac, lung, gastric and airway evaluation and to guide regional anesthesia and vascular access [1].

Ultrasound can be applied to multiple aspects of airway management, such as tube size positioning, predicting successful extubation, guiding cricothyrotomy and predict difficult airway [2]. Several ultrasound parameters have been studied as predictors of difficult laryngoscopy, specifically hyomental distance in neutral and extended position [3], distance from skin to hyoid bone [4], to vocal cords [5], to epiglottis [6], tongue cross-sectional area and volume [7] and many others. Nevertheless, in a recent systematic review and meta-analysis, the most consistent predictor was hyomental distance (HMD) in a neutral position [8].

Since ultrasound is an operator-dependent technique there is a need for structured training and standardized assessment to certify clinician’s skills and competence [9–11]. However, there is no evidence-based guidelines for education or assessment of airway ultrasound.

Traditionally, skill competence was achieved after tutorized clinical training where residents were progressively trusted to practice autonomously by their tutors. The process was complex and was supported by knowledge assessment, gather information from third parties, structured supervision and a direct practical observation of trainees’ performance [12–14]. In the last two decades, many valid and reliable instruments were developed to improve the objectivity of assessment by creating of scales and checklists [15, 16].

In 2013, Tolsgaard and co-workers [17] led an international multispecialty consensus on the content of a generic ultrasound rating scale using a Delphi technique. A total of 60 international ultrasound experts from different medical specialties (radiology, emergency medicine, obstetrics, surgery, urology, rheumatology and gastro-enterology) were invited to participate in three Delphi rounds [17]. Since then, several authors have used this tool to train and assess proficiency for clinical ultrasound in a variety of fields, namely obstetric and gynecology [18–21], abdominal trauma (eFAST) [22, 23], lung [24], head and neck [25] ultrasound.

The Global rating scale (GRS) has been widely used in medical education and in clinical practice as a training and assessment tool. It is usually used as an independent tool or as a complement to support checklists of technical, communication skills or other professional tasks [15, 26, 27]. The present study used GRS as an “overall performance scale” based on 5-points of Likert scale.

We developed an opportunity to train ultrasound hyomental distance measurement for different expertise groups in a simulated environment with standardized patients. The study aims to explore the psychometric properties of the OSAUS scale when used to assess hyomental ultrasound competence.

## Methods

### Materials

#### Ethical approval

The study was carried out following relevant guidelines and regulations. The study was conducted after institutional review committee approval from Ethical Committee for Institute of Life and Health Sciences (CEICVS) of School of Medicine, University of Braga, Braga, Portugal on 15th November 2020 (CEICVS15/2020). Participation in this study was voluntary, and all participants and assessors gave verbal and written informed consent.

#### Study dates

The study was conducted at the School of Medicine, University of Minho, Braga, Portugal, from November 2020 to June 2021.

#### Study design

This is a prospective experimental, rater and principal investigator double-blinded study to determine OSAUS’s psychometric properties when the scale is used for the ultrasound measurement of hyomental distance with the head in neutral position.

**Fig. 1:**
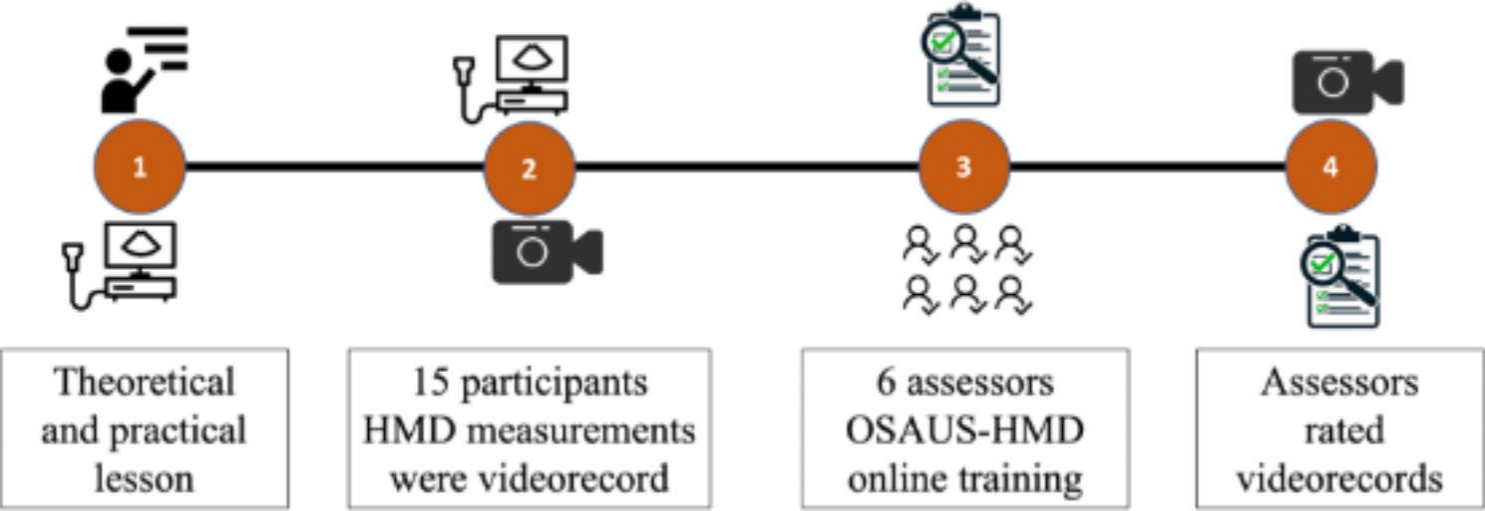
Study design. Step 1 - theoretical presentation and practical session; Step 2 - participants evaluation of HMD in standardized patients, videorecord; Step 3 - assessors’ preparation to use OSAUS scale; Step 4 - evaluation of videorecord participants performance using OSAUS scale.

The study has developed in 4 steps. (Fig. 1) In the first step an educational moment was organized with an one-hour theoretical presentation. Two experienced airway ultrasound anesthesiologists presented the OSAUS scale [17] and its applicability to measuring ultrasound hyomental distance in a neutral position. The protocol for HMD measurement in neutral position was very well-defined and available to consult during the training moment. After this session, a 3-hours of practical training was done with a one-to-one feedback by the experienced trainers. The participants evaluated 9 standardized patients.

Two weeks later, in the second step, participants completed the ultrasound measurement of ultrasound HMD in neutral head position and the performance was video recorded. Each participant evaluated ultrasound HMD in the same three standardized patients, generating 45 videos.

In step 3, six assessors were recruited and received online guidance on the OSAUS scale and its applicability for assessing ultrasound HMD measurement. A concrete preparation analyzing a pilot video was done.

In step 4, assessors blindly rated participants performance according to OSAUS and a global rating scale (GRS) with 5-Likert points (1 point - unacceptable; 2 points – weak; 3 points – acceptable; 4 points – very good; 5 points – excellent performance). The time needed to complete participants’ task was also collected for further analysis. The assessors’ evaluation was sent in an anonymous excel file.

#### Participants

Volunteer participants provided informed consent and self-reported their experience with airway ultrasound before their enrolment. According to participants experience, three categories were created: novices, intermediates and experts. A novice participant had no experience to up to six months of practice in airway ultrasound and expert has used airway ultrasound for more than two years. The intermediate group enrolled participants with experience within six months to two years.

Standardized patients (SP) volunteers: Nine SP were recruited for the study. All SP participated in the training moment and only three of the nine collaborate in the assessment time. All SP were women, between 20 and 25 years old, with healthy weight, a normal to low BMI, and with no previous history of difficult airway or neck deformation or scars. All provided informed consent before participating on the study.

#### Equipment and environment

Steps 1 and 2 were performed at the School of Medicine, University of Minho, and the study equipment and environment were the same for the practical session and for the assessment time. Ultrasound measurements were obtained using a SonoSite®, portable ultrasound machine (Fujifilm, SonoSite® Edge II and SonoSite® SII, Ultrasound System, Inc Bothell, WA, USA), using a curvilinear, multifrequency 3–8 MHz ultrasound transducer probe.

#### Procedure

**Fig. 2:**
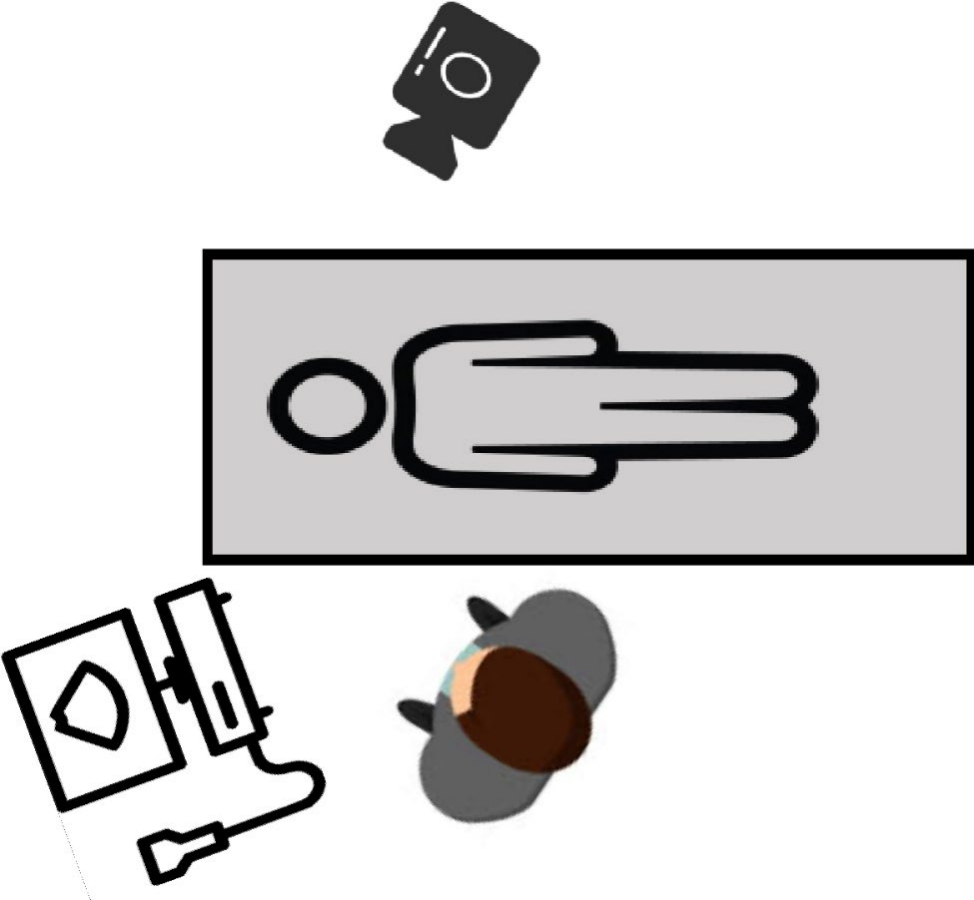
Setting of the ultrasound workstation for training and videorecording. The camera was able to videorecord simultaneously participant’s hands, standardized patient face and neck and the ultrasound screen

Participant measurement of HMD in a neutral position was video recorded. The angle of video records provided a global overview of the technique, including the face of the SP; both hands of the sonographer and all ultrasound machine. (Fig. 2).

Participants were anonymized by not recording their faces or voices. Once the procedure was finalized, the film clips were stored and referenced by order of collection. An anonymous link to a folder with the videos was sent independently to each assessor.

### Methods

#### Psychometric study

The analysis of OSAUS’s psychometric properties was based on the category framework articulated by Messick [28, 29] and according to three domains (internal structure, relation to other variables and response process). The internal structure was analyzed by internal consistency and interrater reliability. The study of the relation to other variables domain included the criterion-related analysis, by comparing [1] OSAUS with GRS and [2] the performance score and [3] time to complete the task from novice-intermedial-expert groups. The response process domain of OSAUS scale evaluation focused on data collection methods; rater instructions, training and performance; how scores were stored and on strategies adopted for the lack of bias in the process [30].

#### Statistical analysis

The internal consistency was assessed through Cronbach’s alpha for each item used (items 2 – Applied knowledge of ultrasound equipment, 3 – Image optimization, 4 – Systematic examination and 5 – Interpretation of images). For each participant, we calculated the mean score of the three ultrasound measurements and the mean time in seconds to complete the task. Intraclass Correlation (ICC) estimates and its 95% confidence interval were calculated. The ICC two-way-random effect model was used to evaluate consistency between raters based on the mean value of the OSAUS score from raters (k = n) [31]. An analysis of variance (ANOVA) for repeated measurements was also done. Convergent validity was assessed by a Pearson´s correlation between OSAUS and Global Rating Scale.

One-way ANOVA explored differences in OSAUS rating scores and differences in time to complete the task between groups with different levels of expertise.

The statistical analysis was done using IBM SPSS Statistics version 29.0.0.0 (IBM Corp, Armonk, NY) with P values below 0.05 were interpreted as the statistical significance and the strength of agreement were interpreted according to Portney [32] where values under 0.5 represent poor reliability, values between 0.5 and 0.69 considered moderate, values between 0.7 and 0.9 indicate strong and over 0.9 represent excellent reliability.

## Results

### General results

#### Participants

Fifteen participants were enrolled on the study, 10 (66.6%) were female, and 5 (33.3%) were male. The mean age of participants was 30±4.6, mean±SD, (min 25, max 39) years old. Each groups received 5 participants.

#### Assessors

A total of 6 assessors were recruited for the study. One assessor was excluded due to an incomplete assessment.

### Psychometric study

#### Internal structure

##### Internal consistency

The scale’s internal consistency was achieved by analysing the use of each item of OSAUS scale (225 = 45 videos * 5 assessors). The internal consistency of OSAUS scale for ultrasound HMD measurement was evaluated with a Cronbach’s alpha of 0.916.

##### Inter-rater reliability

The inter-rater reliability was assessed using the Interclass Correlation Coefficient (model 2,5) when analysed the mean score of the three ultrasound measurements from each participant. The ICC was 0.720 (95%CI 0.408 to 0.893), with a significance level inferior to 0.001 (p < 0.001).

#### Relation to other variables

To explore evidence of validity concerning other variables, we compared OSAUS scale with GRS, and compared the OSAUS scores and time to complete the task across different experience levels (novice, intermediate and expert).

##### OSAUS compared with GRS

OSAUS for HMD measurement was compared with a 5 points-Likert Global Rating Scale (1- poor performance; 5 – perfect performance). The correlation between OSAUS and GRS was studied using the mean score of the three ultrasound measurements done by each participant.

The correlation between OSAUS-HMD and GRS was r = 0.970 (p < 0.001; with 94% of shared variance, r^2^ = 0.941).

##### Group performance comparations

###### OSAUS score

**Fig. 3:**
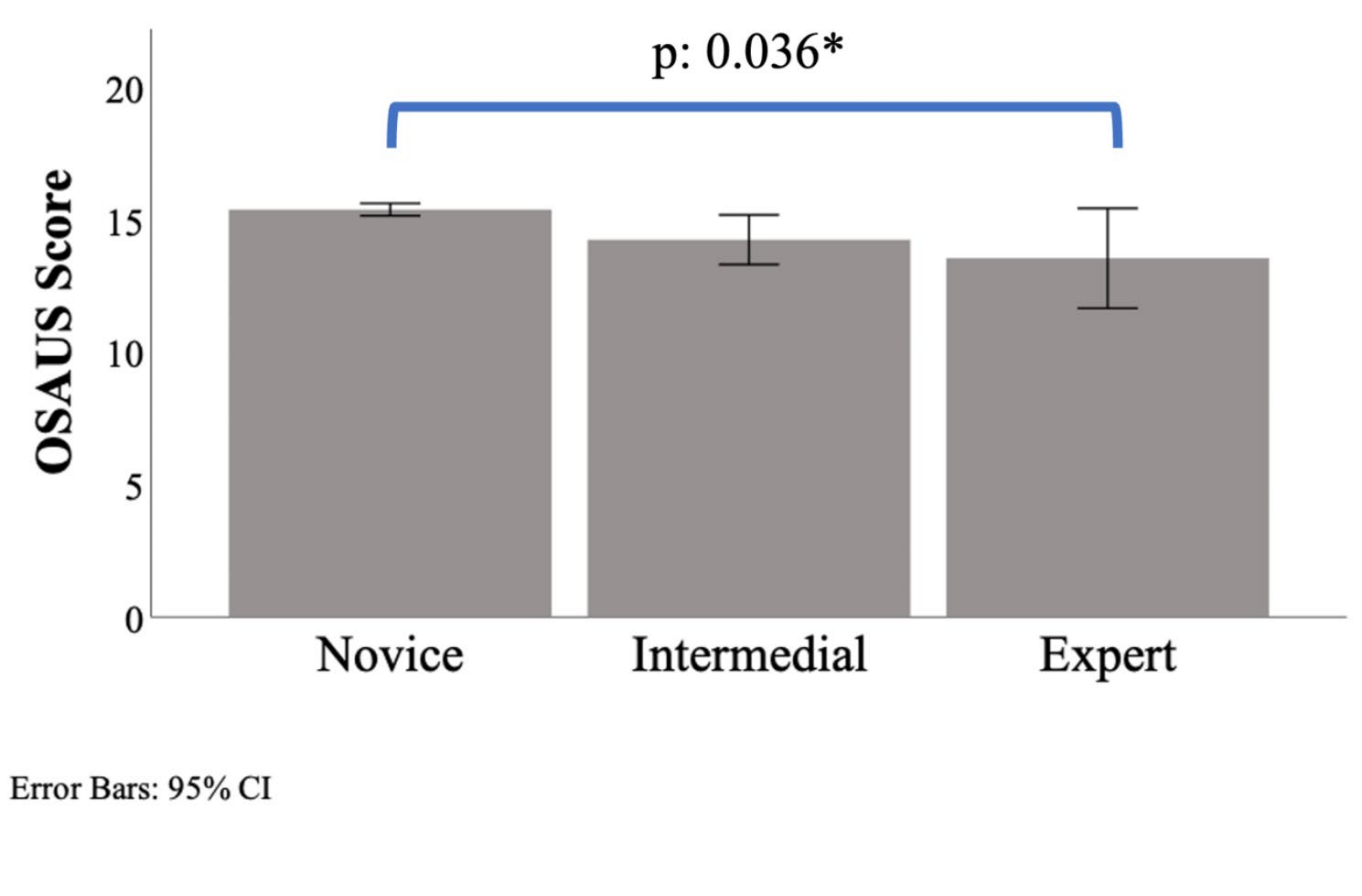
Mean OSAUS scores in each competency group. Values are presented as mean and error bars 95% CI. *One-way ANOVA

The mean value of OSAUS-HMD scores for each group was evaluated considering the mean score of the three measurements from each participant. The novice group scored 15.4±0.18, mean±SD (95%CI 15.1 to 15.6), the intermediate 14.3±0.75, mean±SD (95%CI 13.0 to 14.90) and expert 13.6±0.1.25, mean±SD (95%CI 12.2 to 16.1) (Fig. 3). One-way ANOVA showed significant differences between groups (F(2, 12) = 4.422, p = 0.036). The novice rated higher than the expert group (mean difference of 1.8), with a significant result (p = 0.037, post hoc test, Bonferroni), and higher than intermediate (mean difference of 1.1), with no significant differences (p = 0.279, post hoc teste, Bonferroni). The intermediate group rated a little higher than the expert group (mean difference of 0.7), with no significant differences between groups (p = 0.853, post hoc teste, Bonferroni).

###### Time to complete the task

**Fig. 4:**
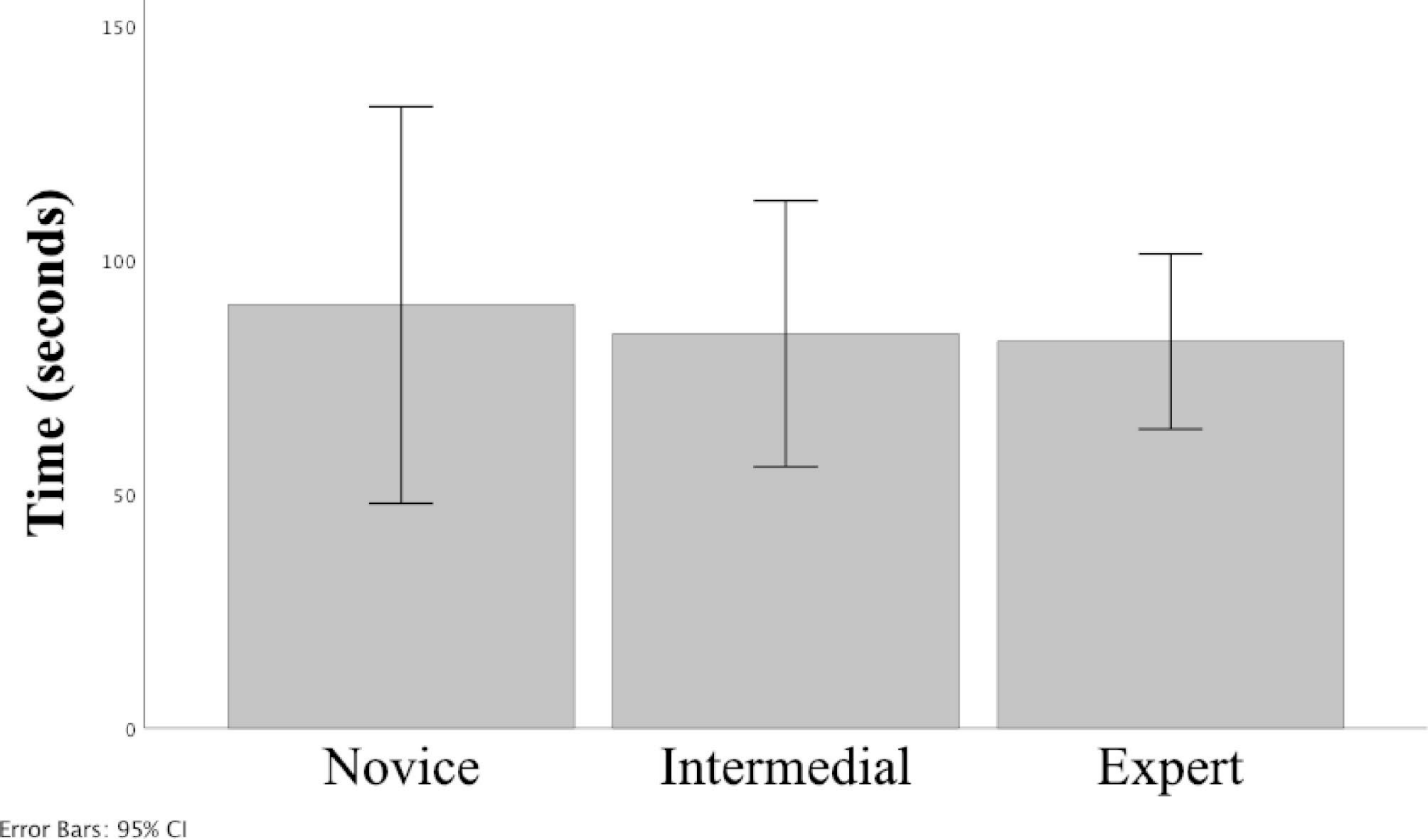
Mean total time in seconds to complete the task in each competency group. Mean and error bars 95% CI for each competency group

In the analysis of the mean time to complete the task for each participant, the novice, the intermediate, and the expert group spent in mean±SD (95%CI) respectively, 90.4±34.2 (95%CI 47.9 to 132.8), 84.2 ±22.8 (95%CI 55.8 to 112.6) and 82.6 ±15.0 (95%CI 63.8 to 101.3) seconds. No significant differences were found between different competency levels (F(2, 12) = 0.133, p = 0.877, one way ANOVA). (Fig. 4)

#### Response process

The participants performances were done individually, with no external interferences, and the video was recorded without participant’s faces or voices. The videos were encoded by acquisition time, which contributed to anonymity. With this approach, we were able to blind the raters.

The rater’s instructions and training consisted of one-hour online session with authors, where the OSAUS scale was presented, and an intermediate performance video was analyzed and rated for training. All assessors agreed to use OSAUS to evaluate participants performance.

## Discussion

The global change for a competency-based medical education extended the need for good assessment tools that could accurately evaluate clinical performance [33–35]. Those tools need to be submitted to a rigorous process of validation before its implementation, so trainees and assessors thrust on the results [36–38]. The present study supports evidence of validity of OSAUS scale, when used to assessed ultrasound HMD, with a strong internal structure, adequate relation to other variables and consistent evidence in the response process domain.

The internal structure measures the “degree to which individual items fit the underlying construct of interest”. In our study its evaluation was based on each time an item was rated (45 videos * 5 assessors = 225 evaluations) [39]. A relevantly excellent internal-consistency was achieved (alfa-Cronbach 0.916) with no evidence of redundant items nor excessive scale length. As the authors excluded three items from OSAUS the internal consistency achieved in this study could be undervalued.

Inter-rater reliability (IRR) was evaluated using interclass correlation coefficient – ICC [2, 5] with a model 2-way model random, since we selected five consistent raters from a larger possible population [31]. IRR was achieved with an ICC of 0.720 (95%CI 0.408 to 0.893), with a significance level (p < 0.001), which reflects a strong agreement between evaluators.

According to the relation to other variables, we compared scores from OSAUS scale with GRS and the ability of the scale to discriminate performance between different groups. We achieved an excellent correlation (r = 0.970, p < 0.001) with Global Rating Scale. OSAUS scale could discriminate between novice and experts. We expected inverted results with experts gathering the highest scores compared with other groups. These results can be secondary to how participants reported their level of competence, and experts were more confident about their level of expertise. Second, the study was developed in a simulated environment away from the clinical reality, where experts were not so familiar with the settings. Nevertheless, they were voluntaries, experts were very difficult to recruit due to their full clinical work agenda and seemed less committed to the study. Those conditions could have compromised their performance. Similar results were obtained from Alvarez-Lopez and co-workers, when used a low-cost 3D portable virtual simulator for skill training in minimally invasive surgery [40]. Simultaneously, the novice participants were at a relatively early stage in their residency program, so that their appetency to learn new techniques and increase their knowledge can explain the higher scores achieved by this group [40]. Although operator-dependent, airway ultrasound skills are easy to acquire, and even the less experienced can achieve performance accuracy with appropriated training. Two recent studies highlighted novice doctors’ ability to quickly learn new technical skills related to ultrasound. Pratheb et al. [41] reported no significant differences in airway ultrasound when comparing novice anesthesia residents with experienced anesthesiologists. Oliveira et al. [42] demonstrated that the learning curve for novices to identify the cricothyroid membrane was relatively short even after a short training of 2 h, with a need of less than 20 scans to achieve competence. The comparation of scores from different competency groups does not represent an essential validity argument [41, 42]. Similarly with the Cook’s study [43] several methodological problems can explain the observed differences between groups not related with the construct of the scale: i) lack of representativity of the population; ii) the novice group was the most homogenous and iii) the group average score does not represent the individual performance.

Methods to obtain evidence about the response process are difficult to develop. A meta-analysis published by Beckman et co-workers [34] and a literature review published by Padilla and co-workers [30] reported the response process as one of the least represented sources of validity in clinical teaching and assessment tools. In our study, a consistent response process was guaranteed by i) assessor’s participation in a training session; ii) assessors’s approvement of the use of OSAUS scale to ultrasound HMD measurement; iii) participants performed individualized, with no external interferences; and iv) adequate quality and security control throughout the steps of the study blinding assessors and authors and reducing the risk for a halo effect.

The authors used the previously developed OSAUS scale to assess ultrasound hyomental distance measurement competence. This approach allowed us to compare our results with previous work and contribute to a boarder application of the scale.

OSAUS scale has been used as an assessment tool for head and neck ultrasonography (HNUS) by otolaryngologists. Todsen et co-workers [25] studied the diagnostic accuracy of surgeon who performed HNUS and stablished the evidence of validity of the OSAUS scale. Similarly with our study, the work recruited a small sample size, used 5 of the 7 items of OSAUS scale. Participants were distributed in two different competence levels (otolaryngologists and interns with no experience in HNUS); and only two raters were invited. This study enrolled patients instead of healthy standardized patients. This approach allowed the authors to establish a correlation between the scale and the diagnostic accuracy. The study presented a good internal structure and discriminated different competence levels.

OSAUS scale was also used to assess competence of ultrasound fetal biometry [18–21]. All studies enrolled participants in two groups (novice and intermedial) according to the number of fetal US. The scale could discriminate the level of competence and the consequence domain was explored with a pass/fail score.

OSAUS scale was used as an assessment tool during the implementation of a simulation training in emergent Focused Abdominal Sonography in Trauma (eFAST) [22] and in transvaginal ultrasound [44]. The scale was also able to discriminate competence group with a good reliability in the assessment of point-of-care ultrasonography.

### Limitations

The study has some limitations that need to be considered the study didn´t explore the content nor the consequences domains of the validity framework from Messick. The content of OSAUS Scale was validated in previous studies [17, 23]. The scale was developed after an international panel of multidisciplinary experts. Three Delphi rounds were necessary to develop the scale, representing a robust concern within its items. The consequence domain of Messick’s framework includes the impact on examinee performance, examinee effects (such as anxiety, stress) and the definition of pass/fail standard [29, 33]. This source of evidence was not explored since the study was done in a simulated environment, far from the workplace and without any professional consequences from a pass/fail evaluation.

The final version of the OSAUS scale has seven elements, with two elements facultative (indication for the examination and medical decision making). Due to the design of this study, we excluded the facultative and the documentation item. The first item - Indication for the examination, is optional and in this study was not evaluated, since all participants were aware of the purpose of the study. The sixth item – Documentation of examination, intends an image recording and a focused verbal or written documentation. It was inappropriate since it could identify participants and consequently bias the results. The seventh item – Medical decision making is also optional and was out of the aim of the study.

Selection of level of competence groups. In our study, participants self-reported their competence level, which might introduce a bias. An external expert panel that could rank participants by level of competency would be more appropriate. The study reported time-of-practice instead of the number-of-procedures criteria to define the three expertise groups. We think that competence in technical skills depends on much more than the number of procedures done, namely on the quality of formative feedback [45]. Nevertheless, experts were voluntary and participated in both moments, their recruitment was challenging, and they seemed to be less engaged with the study when compared with novices and the intermedial group.

Additionally, a portfolio report is not mandatory for consultants, so it would be very difficult to quantify the experience based on numbers instead of self-reported experience. The small sample size is also a limitation.

We use GRS with a single item, as an overall performance scale. Although it was used in order to simplify the process of assessment and to decrease the time spent by assessors to complete the task it could influence the results.

All participants did the assessment moment evaluating a SP already known from the training opportunity. Although the moments were separated by fifteen days, it might improve the scores of all participants, since they were familiar with SP sono-anatomy. Simultaneously, all SP had a thin neck with no dysmorphias. A way to solve this limitation was to consider SP more heterogenous in relation to neck morphology and weight, to assure that participants could do the measurement in both “easy and difficult” necks. This approach could contribute to establishing a pass/fail score and to the process of implementation of the scale in the workplace-based assessment.

## Conclusion

The study demonstrated a strong evidence of validity supporting OSAUS scale to assess HMD competence. The use of OSAUS scale should be integrated into the clinical setting for training and assessment of airway ultrasound competence.

## Data Availability

The datasets generated and/or analyzed during the current study are not publicly available due to the anonymization of the data, but are available from the corresponding author on reasonable request.

## List of Abbreviations

OSAUS: Objective Structured Assessment Ultrasound Skill
PoCUS: Point-of-care ultrasound
HMD: Hyomental distance
GRS: Global rating scale
SP: Standardized patient
IRR: Inter-rater- reliability
ICC: Intraclass correlation Coefficient
US: Ultrasound
